# Demoralization a Source of Disease

**Published:** 1859-10

**Authors:** 


### *Demoralization a Source of Disease.*

Sanitary science has achieved as great triumphs during the last twenty years as any other collateral department of human knowledge and labor. If the application of steam power and of the electric current have abridged time and space—if chloroform has annulled pain, and taken the sting from the sharpest of human pangs—the progress of sanitary science has given increased years to the average space of life, and has driven from the field diseases that were the curse of existence. It is difficult, therefore, to over-estimate the importance of the work which has been done by sanitary reformers, or of that which remains to be done. But it is a great mistake to look at the world exclusively through sanitary spectacles; and into this error many excellent philanthropists now fall. They glance down the columns of the Registrar-General's reports; they read the eloquent figures of Dr. Farr, and his philosophic discussion of the causes that affect the progress of zymotic disease; and from the numberless instances in which death is traced to dirt and neglect, defective ventilation and bad sewerage, they conclude that these are the sole causes of the death-rate that shames our civilization. But indeed we must look further. Let sanitary measures have full play and full encouragement, there will still remain unnatural causes of death which enhance the mortality to a fearful extent.

The most terrible item in the death-rates of great cities is furnished by the account of deaths amongst infants. It varies from half the total mortality to considerably less than one-third. That this great mortality is due wholly to physical conditions, is a theorem too often assumed to be true, but one from which we would emphatically dissent, and which may easily be shown to be false.—For if this were so, then adult life should show an equally striking depreciation of value as the result of hygienic deficiencies. But this is not so. For instance, the city of Glasgow is healthier than London, if the average of the census figures be taken; but the infant mortality is enormous, not less than 53-8 per cent. upon the mortality of all ages. The mortality amongst children is influenced by other conditions besides those of simple hygiene. It is interwoven with the tissue of immorality, of crime, and of neglect, which encircles the population of great towns. There are moral causes for this terrible account of disease and of death. It arises from maternal neglect, from habitual drugging, from desertion, from the absence of medical aid, from the want of food and necessaries, de ied by cruel and unnatural parents. It arises from the dissipation, the intemperance, the improvidence of the laboring classes.

Such a case as that of Michael Croker, indicted for criminal assault upon a girl of thirteen, tried at the Central Criminal Court last week, indicates the nature of that substratum of crime, of misery, and of guilt, which lies at the bottom of a part of our vast infant mortality. Here was a poor girl driven daily from home to beg, to thieve, to wander, to starve; to return home if she could scrape together a sixpence—if not, to lie in the streets. This organized system of cruelty amongst depraved parents is a fertile cause of disease; and, as we value all the means by which disease can be combated and misery arrested, it becomes us to recognize this connexion between vice and physical degradation, and to call earnestly for that moral regeneration which will bring with it material improvement. That also will be a vital reform—a reform that will diminish death-rates, and lessen the doctor's work amongst the masses, which shall have faced and destroyed the vice, the dissipation, the profligacy, the cruelty, and the neglect, which leave children deserted and wives starving, which make girls prostitutes and boys criminals, which make the dark cellar yet more destitute of necessities and more fertile in springs of disease, and which deprive men and women of their moral sense and almost of their human nature. This would also be a sanitary work, although not one of drainage or of water supply; and when we review the accusing figures of the Registrar-General we must bear in mind that both physical and moral causes lie at the root of this great evil.—*London Lancet.*

